# Image Statistics and the Fine Lines of Material Perception

**DOI:** 10.1177/2041669516658047

**Published:** 2016-07-14

**Authors:** Juno Kim, Kairen Tan, Nahian S. Chowdhury

**Affiliations:** School of Optometry and Vision Science, University of New South Wales, Australia

**Keywords:** contours/surfaces, natural image statistics, scene perception, surfaces/materials

## Abstract

We experience vivid percepts of objects and materials despite complexities in the way images are structured by the interaction of light with surface properties (3D shape, albedo, and gloss or specularity). Although the perception of gloss (and lightness) has been argued to depend on image statistics (e.g., sub-band skew), studies have shown that perceived gloss depends critically on the structure of luminance variations in images. Here, we found that separately adapting observers to either positive or negative skew generated declines in perceived gloss, contrary to the predictions of theories involving image statistics. We also found similar declines in perceived gloss following adaptation to contours geometrically correlated with sharp specular edges. We further found this aftereffect was stronger when contour adaptors were aligned with specular edges compared with adaptation to the same contours rotated by 90°. These findings support the view that the perception of gloss depends critically on the visual system’s ability to encode specular edge structure and not image skew.

## Introduction

The visual system neurally encodes images to estimate the surface and material properties of objects, including their 3D shape, lightness, and gloss. Although it has been proposed that the visual system might compute simple image statistics to estimate surface gloss and lightness (e.g., Motoyoshi, Nishida, Sharan, & Adelson, 2007; Sharan, Li, Motoyoshi, Nishida, & Adelson, 2008), other researchers have demonstrated the importance of geometric properties of luminance variations for material perception (Anderson & Kim, 2009; Beck & Prazdny, 1981; Kim, Marlow, & Anderson, 2011, 2012, 2014; Marlow, Kim, & Anderson, 2011; Todd, Norman, & Mingolla, 2004). Here, we examine the viability of image statistics in accounting for perceived gloss in natural images. We also examine the potential dependence of perceived gloss on the encoding of specular edge structure.

Researchers have argued that the perception of gloss depends on computation of simple image statistics—purely photometric information that is independent of image structure (Motoyoshi et al., 2007; Sharan et al., 2008). Motoyoshi et al. (2007) proposed image statistics in the form of histogram or sub-band skew as a model of our brain’s ability to estimate the gloss of surfaces. They found that simply transforming the histogram skew of an image affects perceived gloss, whereas forcing skew to be more positive increased perceived gloss and forcing skew to be more negative reduced perceived gloss. They concluded that perceived gloss depends on the computation of sub-band skew, which they further supported with the finding that adaptation of the visual field to isotropic patterns of positive skew generated declines in the perceived gloss of subsequently viewed surfaces.

Contrary to the Motoyoshi et al. (2007) findings, other researchers have shown that exposure to high-contrast adaptors with no histogram skew also generated declines in perceived gloss (Kim & Anderson, 2010). Kim and Anderson (2010) further demonstrated that adapting to locally dark features (i.e., negative skew) had no effect on perceived gloss and did not increase sensitivity to gloss as predicted by sub-band skew models. They consistently found that the gloss aftereffects were dependent on the proportion of locally bright features contained in the skew adaptors. Based on these results, perceived gloss does not appear to depend on the neural computation of skew, but rather, computations that are sensitive to the contrast of locally bright luminance extrema (i.e., specular highlights). The lack of effect of negatively skewed adaptors might be explained by their photometric incompatibility with the contrast sign of punctate specular highlights generated by the single point light source used in these previous studies.

Rather than depending on the sign of histogram skew or sub-band skew computations, evidence suggests that perceived gloss of surfaces in more natural lighting environments depends on the structure of *specular contours*. Previous studies have shown that generative properties of illumination and 3D surface shape exert constraints on the distribution of specular sharpness and contrast in the image (e.g., Marlow & Anderson, 2013; Marlow, Kim, & Anderson, 2012). Specular sharpness was defined as the steepness of image gradients attributed to specular edges. Indeed, Kim et al. (2012) proposed that perceived gloss depends on the structure of specular “edges,” rather than the brightness of specular highlights per se. Specular edge contours were shown to be generated by not only locally bright specular highlights but also locally dark specular lowlights. Compelling experiences of gloss were generated by simply adding appropriately placed specular lowlights to an image of a diffusely shaded surface.

Computationally, specular edges appear to provide sufficient information to compute the sharpness of specular reflections. The clarity of specular edges—their distinctness of image (DOI)—is one constraint that can be used to estimate the specular lobe of a reflecting surface (Pellacini, Ferwerda, & Greenberg, 2000). Accordingly, research has shown that specular edge sharpness directly influences perceived gloss. For example, blurring specular reflections, reducing their DOI, was also found to reduce perceived gloss (Kim et al., 2012; Pellacini et al., 2000). However, other properties of specular reflectance, such as contrast, appear to provide supplementary sensory information for estimating surface gloss (Marlow et al., 2012). Specular contrast may be computed as the overall local difference between luminance increments and decrements that flank *identified* edge contours attributed to specular reflectance (Marlow & Anderson, 2013). This operation would appear to depend on first classifying image contours as specular edges, reinforcing the importance of detecting specular edge contours.

It would appear that specular edge sharpness, rather than skew per se, might be the primary source of information the visual system encodes to compute surface glossiness. We performed two experiments to evaluate the viability of skew and specular edge sharpness accounts of perceived gloss in a natural lighting environment. Previous studies have used blur to reduce the sharpness of specular reflections, which affects both the amplitude and slope of the luminance profile. Contour adaptation is a method that can be used to selectively reduce visual sensitivity to high spatial frequency edge gradients (Anstis, 2013). Here, we compared the effectiveness of skew and contour adaptation on perceived gloss.

## Experiment 1

To test whether histogram or sub-band skew per se accounts for perceived gloss, we obtained gloss judgments following adaptation to images with isotropic patterns of positive and negative sub-band skew. The skew adaptation model predicts that perceived gloss on local contrast sign; the threshold for perceived gloss should increase following adaptation to positive skew and decrease following adaptation to negative skew. Alternatively, if the structure of specular edges is important for perceived gloss, then adaption to contours aligned with specular edges should generate declines in perceived gloss.

## Method

### Observers

Eight observers participated in this experiment, all of whom had normal or corrected-to-normal vision. All were naïve to the objectives of the study, except for two participants who were authors (K. T. and N. S. C.). Procedures were approved by the Human Research Ethics Advisory Panel at the University of New South Wales.

### Stimuli

**Figure 1.**
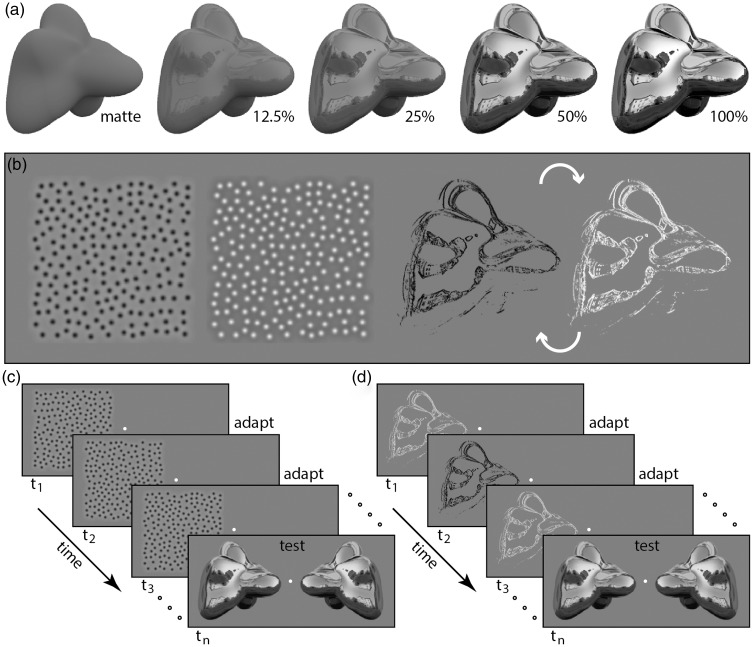
Stimuli and method used for skew and contour adaptation. (a): Rendered images of a 3D object in an outdoor illumination field with specular amplitude increasing from 0 (matte) to 100% according to the Ward (1994) model. (b): Static images showing negative and positive skew adaptors (first and second images, respectively) and contour adaptors. Arrows indicate that bright and dark polarity states of contour adaptors alternate over consecutive frames of adaptation. Example time sequence of adaptation and test on a single trial is shown below, separately for negative skew (c), and contour adaptation trials (d). Central fixation was maintained for approximately 5 s prior to the presentation of rendered surfaces.

We used Blender 3D to render 480 × 480 pixel images of a surface with different levels of gloss (Figure 1(a)). The surface was generated by displacing vertices of a geodesic sphere outward as a function of a Perlin noise map. Images were rendered within a monochromatic, tone-mapped light field, created from an equirectangular image of the Forum in Pisa. We rendered a purely diffuse (lambertian) image and a specular image of the surface. Diffuse and specular components were added together according to a weighted linear combination to generate images of the surface with specular levels ranging from 12.5% to 50% (Ward, 1994). The size of stimuli in test image was approximately 8° visual angle in diameter.

For skew adaptation, we adapted observers separately to positive and negative skew in the form of difference of Gaussian elements identical to those used in a previous study (Kim & Anderson, 2010). Adaptors were ± 2.0 in histogram skew with root mean square contrast of 0.11 and mean luminance of 0.5 grayscale (leftmost pair of images in Figure 1(b)). The gray-level mean luminance of 0.5 corresponded to 39 cd/m^2^. Positively and negatively skewed adaptors differed in that each was the luminance inverse of the other. The peak intensities of adaptors ranged from 2.9 (negative skew) to 197 cd/m^2^ (positive skew). The position of skew adaptors was randomized horizontally and vertically at a rate of 10 Hz. This systematic variation in position was performed to mitigate possible adaptation to edge contours, optimizing adaptation to either positive or negative skewness.

For specular edge adaptation, we used the Anstis (2013) contour adaptation paradigm to selectively and temporarily reduce visual sensitivity to specular edges, but preserving sensitivity to lower frequency surface shading. We applied 3 × 3 Laplacian filtering to the original specular component and then binarized the result to create contour adaptors coinciding with the highest spatial frequency specular edges (rightmost pair of images in Figure 1(b)). Bright and dark contour adaptors were alternated in presentation at a rate of 10 Hz, comparable to the update rate in skew adaptation conditions.

### Procedure

The method of constant stimuli was used to adapt observers to skew (Figure 1(c)) or contours (Figure 1(d)) presented on one side of the display for 5 s. In the no-adaptation control condition, the mid-gray background was presented for the same duration. Test stimuli were displayed immediately after adaptation and were two mirrored images of the same surface geometry presented parafoveally either side of fixation for 1 s (±3°inner-edge separation from fixation). Each test image pair contained a surface with a specular level ranging between 12.5% and 50% and the reference image with a specular amplitude of 25%. Movie 1 (see supplementary online material) closely emulates the display we used to test aftereffects following adaptation to contours (top) and negative skew (bottom), which should have opposite effects on perceived gloss according to skew theory. This demonstration informally shows that following maintained fixation of the yellow point in the top of bottom panels, perceived gloss is experienced to decline.

We verified this effect psychophysically in the present experiment by instructing observers to perform a two-alternative forced-choice task where they maintained central fixation and pressed the left or right arrow button on a keyboard to select the image depicting the surface that appeared glossier. Presentations were randomized across conditions and counterbalanced across the display, totaling 280 trials performed by each observer (4 Adaptor Conditions × 7 Specular Amplitudes × 10 Repeats).

### Statistical Analysis

We estimated the probability a surface with a given level of specular amplitude was selected as glossier than the reference surface. Psychometric curves were fit to probabilities of perceived gloss as a function of specular amplitude using a Weibull function as in a previous study (Kim & Anderson, 2010). The curves were used to estimate the point of subjective equality (PSE), the threshold at which increasing specular amplitude generated probabilities of perceived gloss that were above 50% chance. We used a repeated-measures one-way analysis of variance (ANOVA) and a series of follow-up *t* tests to analyze planned contrasts to test for differences in mean PSE across conditions.

## Results and discussion

**Figure 2.**
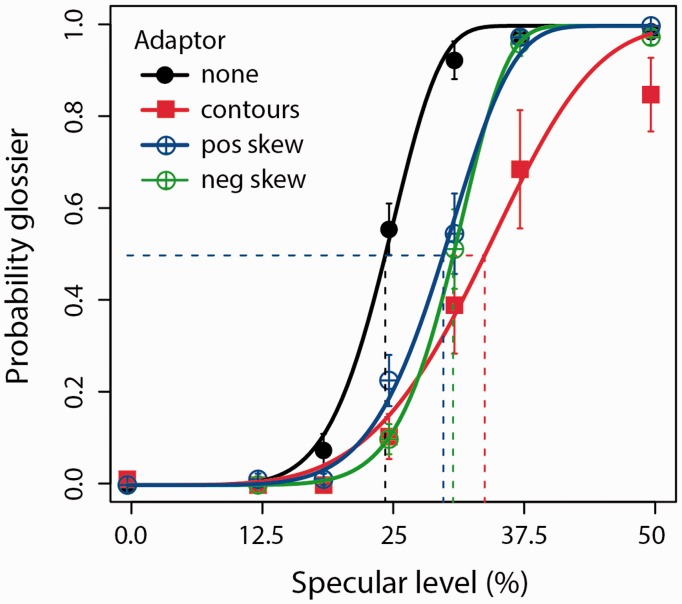
Probability estimates of perceived gloss plotted as a function of specular amplitude. Black: no-adaptation control; Red: aligned contour adaptors; Blue: positively skewed adaptors (pos skew); Green: negatively skewed adaptors (neg skew). Dashed lines indicate specular level at PSE. Error bars show standard errors of the mean, based on data obtained from the eight observers.

Figure 2 shows the result of adapting to positive and negative skew, in addition to contours. There were rightward shifts in PSE across all adaptation conditions, indicative of elevated thresholds for perceived gloss. A one-way ANOVA found a significant difference in PSE across adaptation conditions (*F*(3, 21) = 7.50, *p* < .005). A follow-up *t* test found a significant difference in PSE following adaptation to contours, compared with the no-adaptation control (*t*_7_ = 3.43, *p* < .05). Compared with the no-adaptation control, the PSEs for skew adaptation were both increased following adaptation to either positive skew (*t*_7_ = 5.26, *p* < .005) or negative skew (*t*_7_ = 10.77, *p* < .00005). There was no significant difference between the PSEs following adaptation to positive and negative skew (*t*_7_ = 1.60, *p* = .15).

Finally, we also compared the PSEs for perceived gloss between contour conditions and the skew adaptor conditions. The PSE for the contour adaptation condition was not significantly greater in comparison to the PSE for both the positive skew condition (*t*_7_ = 1.71, *p* = .13) and the negative skew condition (*t*_7_ = 1.54, *p* = .17). The similarity in PSEs suggests that contour adaptors were just as effective as skew adaptors in lowering observer sensitivity to perceiving gloss.

Contrary to image-statistics accounts, these findings suggest perceived gloss does not depend on the detection of contrast sign or photometric brightness of specular reflections. Instead, the gloss aftereffect following contour adaptation suggests perceived gloss depends on visual sensitivity to specular edge structure. On presenting four naïve observers with Movie 1 following their participation, all indicated that the specular reflections following both contour adaptation and negative skew adaptation appeared to have less overall contrast, compared with test surfaces on the nonadapted side of the display. This is consistent with the interpretation that perceived specular contrast, and not just sharpness, depends on the encoding of specular edge structure. Although there was a difference in the effect size (*t* values) between contour and skew adaptation, this difference could reflect the dependence of contour adaptation on effective fixation, which was not as essential during larger-field skew adaptation.

## Experiment 2

Experiment 1 found that perceived gloss could not be explained by the computation of image skewness. Adaptation to local contrast and not contrast sign (i.e., skew) was found to modulate the perception of gloss. In contradistinction, contour adaptation generated strong declines in perceived gloss consistent with the view that sensory coding of specular edges is critical for computing surface gloss. If this effect depends on adaptation to the structure of specular edges, then the effect should weaken when adapting observers to contours rotated out of alignment with specular edges in test images. Hence, Experiment 2 examined whether the observed effects depended on the alignment of contour adaptors with luminance edges generated by specular reflectance and not just on their spatial and temporal flicker frequency properties.

## Method

### Observers

Nine observers participated in Experiment 2, one of whom was an author (N. S. C.). All procedures adhered to the ethical principles of the University of New South Wales and the Declaration of Helsinki.

### Stimuli

Contour adaptors were identical to those used in the previous experiment. However, we used seven levels of specularity ranging up to the full specular amplitude of 100% (12.5%, 25.0%, 37.5%, 50.0%, 62.5%, 75.0%, and 100.0%). This range increase was imposed in order to make the task of parafoveally judging differences in gloss easier for the observers.

### Procedure

**Figure 3.**
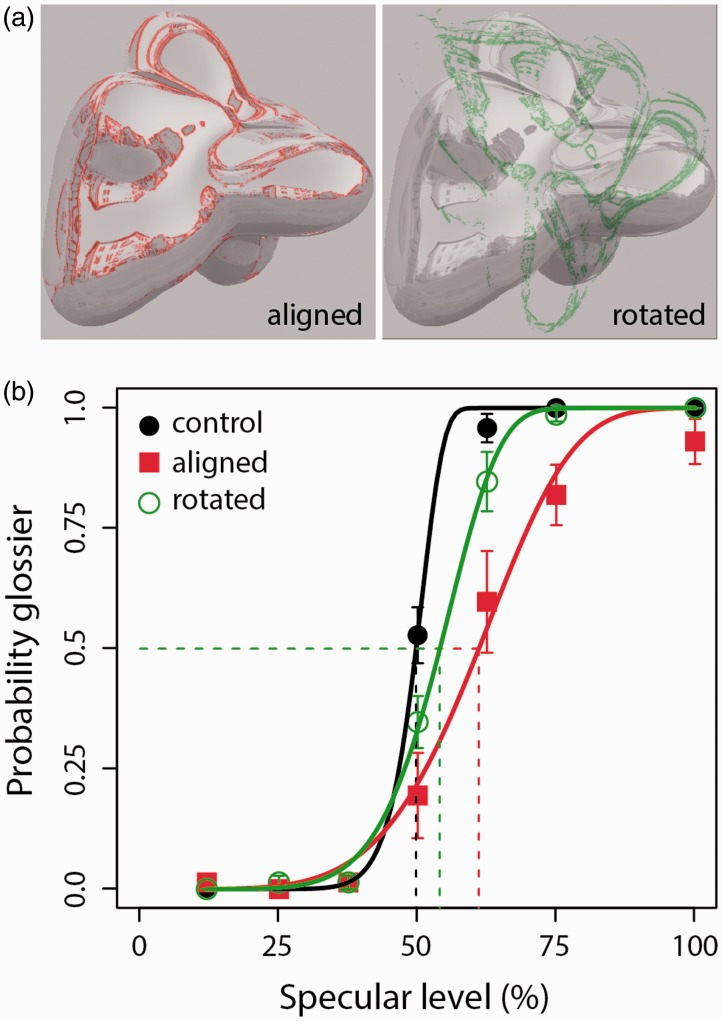
Adaptors and results show effect of contour orientation on perceived gloss. (a): Contour adaptors were either aligned (emphasized in red) or rotated clockwise by 90° (emphasized in green) relative to subsequently presented surface images. Black: no-adaptation control; Green: rotated contour adaptors; Red: aligned contour adaptors. Dashed lines indicate specular level at PSE. Error bars show standard errors of the mean, based on data obtained from nine observers.

The adaptation method was the same as the two-alternative forced-choice task used in Experiment 1. We compared perceived gloss following adaptation to either aligned contours or contours rotated clockwise out of alignment with specular edges by 90° (as shown in Figure 3(a)). This experiment directly tested whether the adaptation effects critically depend on the congruence of adapting contours with specular edges.

## Results and discussion

Figure 3(b) plots the psychophysical data obtained following adaptation to aligned and rotated contours. A one-way ANOVA found a significant difference in mean PSE for perceived gloss across conditions (*F*(2, 16) = 8.61, *p* < .005). A follow-up *t* test found no significant difference in PSE following adaptation to rotated contours, compared with the no-adaptation control (*t*_8_ = 1.95, *p* = .09). However, there was a significant difference in PSE following adaptation to aligned contours, compared with adaptation to rotated contours (*t*_8_ = 2.96, *p* < .05) and the no-adaptation control (*t*_8_ = 3.14, *p* < .05). Following their participation, three naïve observers were asked to describe the appearance of a surface with 12.5% specular level after exposure to aligned contour adaptors. All reported that the environmental reflections appeared lower in clarity, compared with the same image in the unadapted visual field.

## General Discussion

We primarily sought to assess whether sub-band skew models could account for perceived gloss in a natural illumination field. The ineffectiveness of sub-band skew computations was confirmed by the finding that adaptation to positive and negative skew both generated declines in perceived gloss (Experiment 1). We further found that selectively reducing sensitivity of visual cells with receptive fields tuned for sharp specular edges generated declines in perceived gloss. This finding is supported by the dependence of perceived gloss on the alignment between contour adaptors and specular edges. Consistent declines in perceived gloss were observed following adaptation to contours aligned with specular edges and were not observed following adaptation to contours rotated out of alignment with specular edges (Experiment 2). These results cannot be explained by differences in statistical properties of the adaptors, as the underlying image statistics were preserved across changes in the orientation of these adaptors.

The similar threshold increases following adaption to positive and negative skew is consistent with the view that perceived gloss depends on coding specular contrast per se, rather than image statistics in the form of histogram or sub-band skew. The original Motoyoshi et al. (2007) proposal was that perceived gloss is directly correlated with the sign of an encoded image’s histogram skew. According to this view, adapting to positive skew should reduce sensitivity to perceiving gloss (i.e., increase PSE) and adapting to negative skew show increase sensitivity to perceiving gloss (i.e., decrease PSE). Hence, the aftereffect should be to some degree symmetric. Contrary to this view, however, previous research showed that adaptation to negative skew had no effect on perceived gloss, presumably because the only available cues to specularity available in the Motoyoshi et al. (2007) stimuli were locally bright specular highlights (Kim & Anderson, 2010). The current study avoided this limitation by using images of glossy surfaces where the specular component was rendered in a natural outdoor light field. This light field generated specular edges defined not only by locally bright specular highlights but also locally dark specular lowlights. Critically, we found that adapting to negative skew *decreased* perceived gloss (increased PSE), contradicting the prediction of image statistics that such adaptation should increase sensitivity to perceiving gloss. The effect of these adaptors suggests the skew aftereffects depended on the coding of specular contrast per se and not sub-band skew.

The findings together support the alternative view that perceived gloss depends critically on the visual system’s ability to encode information about the sharpness of edges generated by a surface’s specular reflectance (Kim et al., 2012; Pellacini et al., 2000). Previous work showed that simply blurring the specular component of an image decreases perceived gloss, presumably due to imposed changes on specular sharpness—the DOI (Pellacini et al., 2000). Kim et al. (2012) found that such blur decreased perceived gloss when only applied to specular lowlights, highlighting the importance of specular edges for the computation of gloss and not the brightness of reflections.

It should be emphasized that the sharpness of reflections alone is not the only important source of information for perceiving gloss. The structure of the light field and 3D shape influence the contrast, coverage, and sharpness of specular reflections (Marlow & Anderson, 2013; Marlow et al., 2012). These image constraints generate distinctly different perceptual attributes. Although perceived specular sharpness correlates well with perceived gloss (Marlow et al., 2012), encoded sharpness has the potential to modulate the experience of specular contrast. This dependency is supported by the current findings and the consistent observation that following adaptation to aligned contours, the specular component temporarily appears to decline in both clarity and contrast (Movie 1). This decline in *perceived* global contrast of the entire specular component is likely to be attributed to an integrative mid-level process. Similar adaptation to contours is also known to suppress awareness of variations in lightness across an edge, which was attributed to the effect of contour adaptors on the sensitivity of orientation-selective V1 cells (Anstis, 2013). These findings would appear to suggest that image contours provide a critical source of information the visual system must encode for the mid-level computation of material properties.

It should be noted that the specular reflections in our displays were always geometrically compatible with diffuse shading, satisfying image constraints for the classification of edges as specular. Previous studies have shown that attribution of contours to gloss, texture or occlusion depends on their geometry relative to surrounding shading (Beck & Prazdny, 1981; Kim et al., 2011, 2012, 2014; Kim and Anstis, 2016; Marlow et al., 2011; Todd et al., 2004). However, the presence of nonspecular (diffuse) shading is not essential for the perception of gloss; the perceptual attribution of luminance gradients to gloss readily succeed in the absence of diffuse shading, where compelling experiences of gloss and shape are generated by the structure of specular reflections alone (Fleming, Dror, & Adelson, 2003; Fleming, Torralba, & Adelson, 2004; Norman, Todd, & Orban, 2004).

In conclusion, we find evidence to suggest that image statistics, in so far as they do not consider the structure of luminance variations in an image, do not explain the perception of gloss. The success of our contour adaptors suggests that perceived gloss depends critically on the coding of specular edge structure. It could be argued that because contour adaptors stimulate both low-level and mid-level visual circuits nonspecifically, it is difficult to discern their relative contributions to the gloss aftereffects observed. However, the physiological affinity of orientation-tuned simple cells in V1 for contour adaptation seems to be a likely account for these gloss aftereffects (Anstis, 2013). The implication of mid-level processing was also supported by the demonstrated decline in perceived *global* specular contrast (e.g., Movie 1). Future research will hopefully provide insight into the mid-level processes underlying the perception of specular contrast and gloss, including scenarios where diffuse shading is absent.

## Supplementary Material

Supplementary material

## Supplemental Material

All supplemental material mentioned in the text, including three figures, is available in the online version of the journal at http://IPE.sagepub.com/supplemental
