# CORRIGENDUM to Image Statistics and the Fine Lines of Material Perception

**DOI:** 10.1177/2041669516686457

**Published:** 2016-12-01

Juno Kim, Kairen Tan, andNahian S. Chowdhury (July-August 2016). Image Statistics and the Fine Lines of Material Perception. *i-Perception*, 7(4), 1–11. (DOI: 10.1177/2041669516658047).

A change has been made to this article after its publication on July 14, 2016 issue of i-Perception. These corrections will be included in all subsequent versions of the article online.

In the **Author Biographies** section, the image for author Kairen Tan was omitted and the biography should read as follows:

**Figure:**
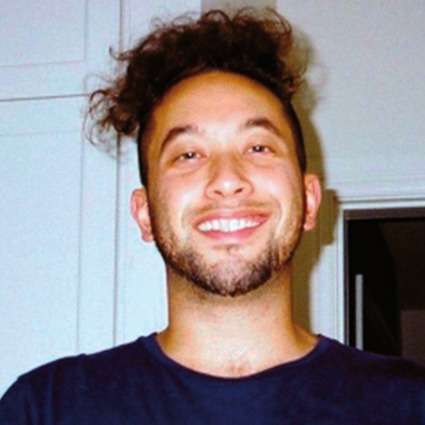


**Kairen Tan** is a graduate student with a Masters of Information Technology at the University of New South Wales. His research uses image processing and analysis to understand the perception of surfaces and materials in real-world scenes.

